# Towards a Dynamic Model of the Kangaroo Knee for Clinical Insights into Human Knee Pathology and Treatment: Establishing a Static Biomechanical Profile

**DOI:** 10.3390/biomimetics4030052

**Published:** 2019-07-25

**Authors:** Manaal Fatima, Corey J. Scholes, Emily Zhong, Lawrence Kohan

**Affiliations:** 1 EBM Analytics, Crows Nest, NSW 2065, Australia; 2 Joint Orthopaedic Centre, Bondi Junction, NSW 2022, Australia

**Keywords:** patellofemoral pain, biomechanical profile, kangaroo knee, biomimetics

## Abstract

There is limited understanding of how patella realignment or patellectomy to surgically manage patellofemoral pain (PFP) affects knee biomechanics. By analysing marsupials like kangaroos that lack an ossified patella, actionable biomimetic insight for the management of end-stage PFP could be gained. This study aimed to provide the foundation of a multi-stage approach, by establishing a static biomechanical profile of the kangaroo stifle that informs the inputs and factors requiring consideration for future dynamic analyses. Volumetric CT and MRI sequences were obtained for four hindlimbs from two *Macropus giganteus* specimens, from which three-dimensional models of the stifles were created. Two limbs were dissected to visualise the insertion points, origins and lines of action of the quadriceps muscles and the knee extensor mechanism. Static measurements were obtained from the three-dimensional models to establish the biomechanical profile. The results confirmed structural differences in the kangaroo stifle with lack of an ossified patella, a prominent tuberosity and a shorter femur, which functionally affect the mechanical advantage and the torque-generating capability of the joint. The data reported in this study can be used to inform the inputs and constraints of future comparative analyses from which important lessons can be learned for the human knee.

## 1. Introduction

Patellofemoral pain (PFP) is one of the most prevalent knee disorders and is associated with pain and functional limitations in affected patients, who report a reduced quality of life [1,2]. Growing evidence suggests that PFP lies on a continuum with patellofemoral (PF) osteoarthritis (OA), and is driven by factors such as altered biomechanics, tissue homeostasis, and non-mechanical changes in the PF joint [3]. Despite the prevalence of PFP, questions remain surrounding the underlying pathology, associated risk factors, biomechanical relationship with OA, and the best course of management.

Surgical interventions to manage PFP typically involve realignment of the extensor mechanism to address patella maltracking and instability, with tibial tuberosity (TT) medialisation and anteromedialisation to correct a laterally misaligned patella [4]. In PFP, there is elevated pressure and shear stress at the patella and femur chondro-osseous interface [5], which TT medialisation of about 10 mm decreases by up to 15%, without overloading the medial cartilage [6].

Patellectomies (total or partial) are uncommon and are reserved as a salvage procedure for severe fractures or more advanced cases of OA, infections and tumoral conditions [7]. They significantly affect the biomechanics of the knee extensor mechanism, resulting in ligament instability, reduced muscle strength and loss of range of motion [8,9]. Tensioning of the knee extensor mechanism, to compensate for the reduced lever arm of an absent patella, has been reported with good outcomes at 11-year follow-up in young patients [10]. However, information remains lacking in the literature regarding the mechanics of the extensor mechanism, and correct loading of forces in the PF joint following realignment or removal of the patella to manage PFP.

The key function of the patella is to modify the mechanical advantage at the knee joint by increasing the moment arm of the quadriceps tendon in which it is embedded and decreasing the quadriceps force required to generate an adequate extension moment. Interestingly, most marsupials and many reptiles lack an ossified patella [11], likely related to the preferred locomotion pattern of each species [12]. Nevertheless, comparing biomechanical parameters, such as muscle moment arms and segment dimensions, between species may yield insights into the evolution of selected species. Furthermore, an understanding of the structural and functional differences, and evolutionary adaptations of species lacking a patella, could provide actionable biomimetic insight for the management of end-stage PFP with patellectomy.

Kangaroos are bipedal at higher locomotion speeds and are an example of a marsupial lacking an ossified patella. In its place, there exists a fibrocartilage pad in the insertion tendon of the quadriceps femoris muscle [13]. While there may be lessons for the treatment of human knee pathology within the stifle of the kangaroo, a biomechanical comparison of the two species has presently only been explored with respect to mechanical limits on running ability [14]. A comparison of the structural and functional differences between the human and kangaroo knee may provide further insights into the mechanics of the extensor mechanism within the context of an absent patella; however, such comparisons will require a dynamic assessment of knee biomechanics throughout a range of motion [15]. Given the complexity of the knee joint, development of a static model of the kangaroo in the first instance, will enable the establishment of accurate boundary conditions for a dynamic analysis [16] and confirm the viability of the static biomechanical profile for dynamic analysis [17].

In order to obtain meaningful lessons from the kangaroo knee that can be applied to the human context, this study aims to present a static biomechanical profile of the kangaroo stifle, with particular focus on the extensor mechanism, its moment arm, limb lengths and the profile of the quadriceps muscles, from which quantifiable metrics for consideration in future dynamics analyses can be derived.

## 2. Materials and Methods

### 2.1. Specimen Collection

Four hindlimbs from two specimens professionally culled for damage mitigation purposes (eastern grey, *Macropus giganteus;* one female and one male) were sourced from Queensland, Australia. The available limbs were partly degloved and had no pelvis, but the femoral head through to the end of the tibia were intact. The specimens were fresh frozen on site and transported to Sydney, Australia. All limbs were kept frozen in sealed bags to prevent freezer burn in an animal storage facility. Approval from an Animal Ethics Committee (AEC) was considered but deemed not required for this research as the study did not impact any aspect of the kangaroo’s life or death (confirmed by the AEC at the University of Technology, Sydney). 

### 2.2. Specimen Imaging

**Figure 1:**
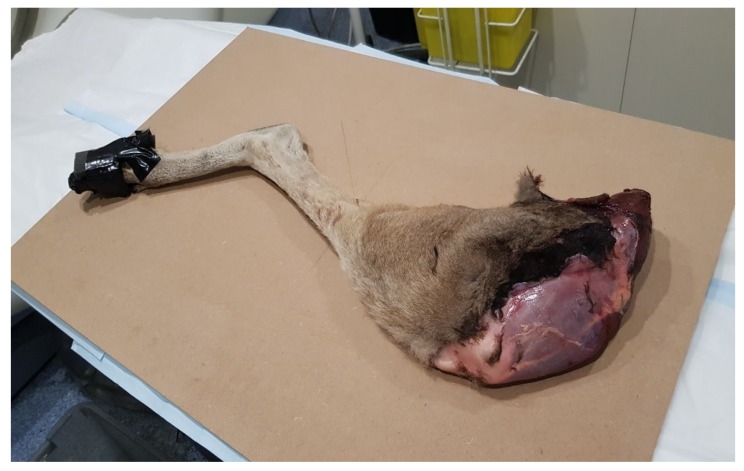
A defrosted limb ready for imaging. All limbs had the femoral head to end of tibia intact and were partly degloved (as sourced).

The specimens were defrosted for 72 h and removed from their bags prior to scanning (Figure 1) at a clinical imaging facility (Castlereagh Imaging, Cremorne, Sydney, Australia). MRI sequences were obtained from a Philips Ingenia 3.0T scanner (Philips Medical Systems, Amsterdam, Netherlands). The scanning protocol included two sequences: (1) a three-dimensional proton-density high resolution sequence (TR—1000 ms, TE—37.7 ms, in-plane resolution of 1024 × 1024 pixels, 0.52 mm slice thickness and 0.26 mm spacing; and (2) a two-dimensional proton-density sequence captured in the coronal plane to the thigh segment (TR—2955 ms, TE—30 ms, in-plane resolution 960 × 960, 2.5 mm slice thickness and 2.75 mm slice spacing. In addition, CT sequences were obtained from a GE Medical Systems LightSpeed VCT scanner (LightSpeed VCT, GE Healthcare, Chicago, IL, USA) with 512 × 512 resolution, 0.63 mm slice thickness, 0.3 mm slice spacing and 120 pkV.

### 2.3. Specimen Dissection

An orthopaedic surgeon performed the dissection on two limbs using a standard dissection kit, starting from the superficial layers and progressing systematically to the femur laterally via the iliotibial band and intermuscular septum of the vastus lateralis (VL) [18]. The knee extensors and flexors were identified, with a particular focus on the insertion points, origins and lines of action of the rectus femoris (RF), sartorius (SA), vastus intermedius (VI), and the VL. The specimens were repositioned onto their lateral side, with the medial aspect uppermost to allow access to the vastus medialis (VM). The tibial attachments were identified and key features marked with pins. The specimens were photographed to assist with imaging analysis.

### 2.4. Modelling the Bone and Extensor Mechanism

**Figure 2:**
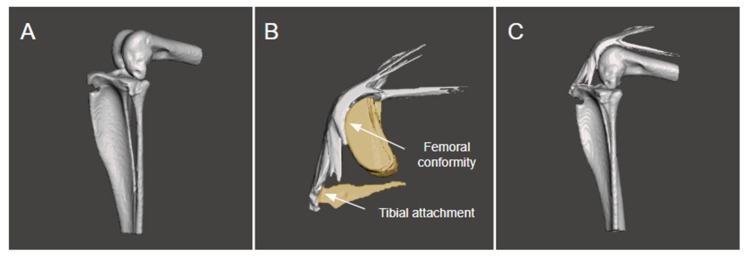
Three-dimensional models of the femur and tibia (**A**) were assembled with the knee extensor mechanism (**B**) using the femoral conformity and tibial attachment as a point of reference to generate the final model (**C**).

DICOM files were processed using a custom-written MATLAB script (Mathworks, v9.4 (2018a); Mathworks, Natick, MA, USA) for sorting into sequence order. Segmentation of the tibia and femur was performed using Seg3D (v2.4.3, Scientific Computing and Imaging Institute, University of Utah, Salt Lake City, USA) through manual titration of upper and lower threshold limits to adequately capture bone on each CT. The Segmentation Level Set filter was applied on the thresholded area using the default settings. The stifle extensor mechanism was manually segmented in Seg3D on each sagittal slice of the MRI scans using the Polyline tool for segmentation of complex shapes, with the femoral conformity and tibial attachment segmented for reference. Three-dimensional models of the tibia and femur and the extensor mechanism were generated and isosurfaces exported as an STL file for further smoothing using 3D sculpting-based CAD software (Meshmixer v3.4.35, Autodesk Research, San Francisco, CA, USA). The bone (Figure 2A) and tendon (Figure 2B) models for each of the four limbs were imported into a 3D modelling software (Rhino v6, Robert McNeel and Associates, Seattle, WA, USA) and individually aligned using the femoral conformity and tibial attachment reference points for guidance (Figure 2C). A total of four joint models, one for each specimen limb, were assembled. 

### 2.5. Static Measurements

Limb segment profiles were established using anatomical landmarks on the three-dimensional models. Femoral length was measured on the sagittal plane as the distance between the greater trochanter of the femur and the intercondylar notch [19]. Tibial length was measured on the coronal plane as the distance between the tibial plateau (TP) and the lateral malleolus. The flexion angle was calculated using the law of cosines as the angle between the midshaft of the femur and the midshaft of the tibia in the sagittal plane.

**Figure 3:**
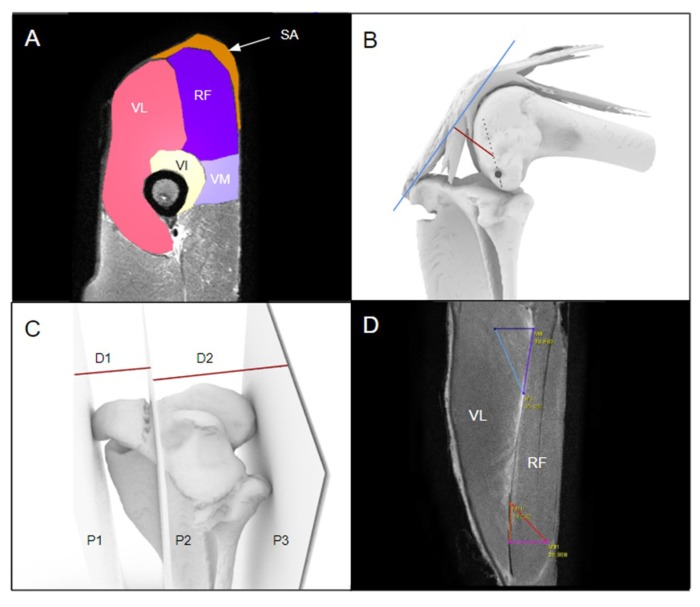
Static characteristics of the kangaroo stifle were derived from the 3D models by measuring: (**A**) The muscle cross-sectional areas, with the VL, VI, VM, RF and SA labelled; (**B**) the moment arm, shown in red. The grey dot marks the sulcus where the lateral collateral ligament attached, and the grey dotted line represents the axis of rotation of the femoral condyles. The plane representing the line of action of the extensor mechanism is shown in blue; (**C**) the TP:TT ratio and the TT projection index, and (**D**) the muscle pennation angles of the VL and RF.

The muscle cross-sectional area was calculated by segmenting the main muscles in the coronal plane around the midlength of the femur, midway between the greater trochanter of the femur and the articular cleft between the condyles of the femur and the tibia [19]. Approximately 10 slices of the middle of the muscle belly were segmented in Seg3D and the average cross-sectional area across the slices was calculated for each specimen (Figure 3A). The cross-sectional area of the RF, VL, VI and WM was summed to give the total quadriceps cross-sectional area (QCA). The QCA was multiplied by the length of the femur (FL) to approximate the muscle volume of the quadriceps femoris [19]. 

The moment arm was measured on the assembled models using two key lines of reference (Figure 3B). The first was the axis of rotation of the femoral condyles, defined as the transepicondylar axis between the collateral ligament attachment points on the femoral condyles, observed as sulci medially and laterally. The second was a plane to represent the line of action through the extensor mechanism. The moment arm was measured as the perpendicular distance between the extensor mechanism line of action and the transepicondylar axis (Figure 3B). 

The ratio of the TP to the TT (TP:TT ratio) was measured by creating three parallel planes on the tibial model (Rhino v6, Robert McNeel and Associates, Seattle, WA, USA) (Figure 3C) at the most anterior point of the TT (P1), at the anterior cortex of the tibia (P2), and at the posteriormost part of the TP (P3). The perpendicular distances between the planes were measured, with the distance between P1 and P2 recorded as D1 and the distance between P2 and P3 recorded as D2. The TP:TT ratio was calculated by dividing D2 by D1. The TT projection index was calculated as the length of TT relative to tibial length (D1 divided by tibial length) [20]. 

Muscle pennation angle of the quadriceps was calculated by selecting the main muscle belly of the VL and RF, and creating a straight line in the axial plane along the aponeurosis. The muscle striations were visually identified and a line drawn parallel to them to intersect the aponeurosis axis (Figure 3D). The lines were resized to create a triangle, and the pennation angle calculated using the law of cosines [21]. 

## 3. Results

### 3.1. The Extensor Mechanism

**Figure 4:**
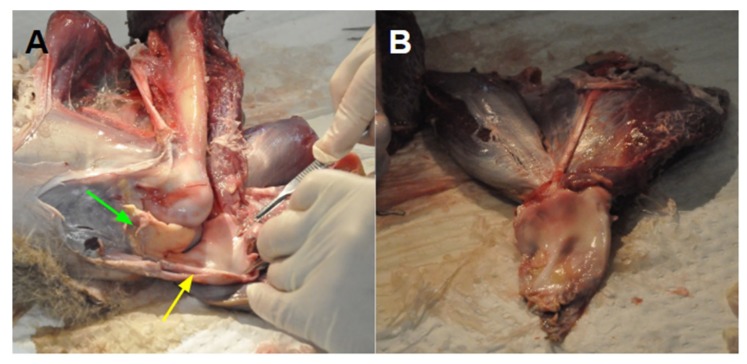
(**A**) Extensor mechanism (yellow arrow) pulled away from the femoral head, revealing a fat pad underneath (green arrow). (**B**) Cuff-like thickening with tibial attachments removed.

The specimens lacked an ossified patella, and in its place was a fibrocartilaginous pad, resembling that of a shoulder rotator cuff. The thickening conformed closely to the surface of the femur, with a fat pad underlying the attachment of the extensor mechanism to the tibial tuberosity (Figure 4). It was noted that there was no attachment of the extensor mechanism directly to the femur.

### 3.2. Static Measurements of the Kangaroo Stifle

The static measurements of the kangaroo specimens measured from the 3D models are summarised in Table 1. The median was taken as a representative measure given the large range and the small sample size.

### 3.3. Muscle Attachment and Insertion Points

**Figure 5:**
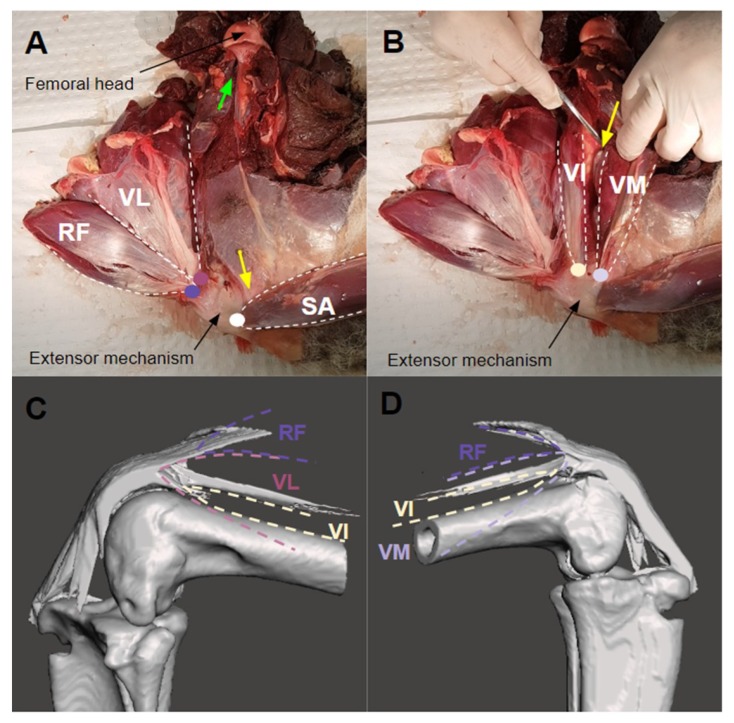
(**A**) Medial view: SA reflected from the RF to visualise the deep muscles underneath. Extensor mechanism insertion of the VM marked by the yellow arrow, and the femoral attachment point of the VM marked by the green arrow, originating from the lesser trochanter. (**B**) VM sits superficial to the VI. Medial intermuscular septum (yellow arrow) separates the VM and VI. (**C**) Lateral and (**D**) medial views of the representative insertions of the RF, VL, VI and VI muscles. Colour coded insertion points are also marked on (**A**,**B**).

The dissection established the key extensor anatomy of the kangaroo lower limb. Muscle origin points, insertion points into the extensor mechanism, and femoral attachments to gauge lines of action are summarised in Table 2. The SA was superficial to the quadriceps muscles, and the RF attached anteriorly to the VL. The origin points of the SA and RF were unavailable in the specimens but were verified in the literature to originate from the tuber coxae and ilium respectively [18], and the VM and VI were found to originate from the lesser and greater trochanter respectively (Figure 5A).

The medial intermuscular septum separated the VM and VI, and the VM attached to this septum, not the femur (Figure 5B, green arrow). The VI attached laterally down the femur from the greater trochanter to about 3 cm from the end of the femur, and part of the VL attached anteriorly to the VI. The VL had no attachment to the femur, with a single tendon of origin attached to the greater trochanter. The representative insertions of the muscles into the extensor mechanism are shown in Figure 5C,D.

## 4. Discussion

The aim of this paper was to establish a static biomechanical profile of the *M. giganteus* stifle with exploration of the structural characteristics of the extensor mechanics, in order to inform the inputs and factors that need to be considered for future dynamic analyses. We specifically report on limb segment profiles, muscle profiles, and the extensor mechanism profile with respect to the moment arm in the kangaroo stifle. The findings confirm structural anatomy in the kangaroo knee with lack of an ossified patella, increased projection of the TT and a shorter femur, and associated functional implications, most notably affecting the mechanical advantage and the torque generating capability of the knee joint. 

### 4.1. Structural Characteristics

The combined dissection and high-resolution imaging analysis confirmed that kangaroo stifles differed to humans in a number of key characteristics. Firstly, ossified patellae were lacking in the four limbs sourced from two specimens, with a cartilaginous thickening and fat pad in its place, consistent with previous descriptions [13] and in contrast to the adult human knee. The lack of patella may have had an impact on the length of the moment arm, with the present results (~29 mm at an average flexion angle of 74°) much lower than previously reported for human adult cadaver specimens (~50 mm) [22] and in-vivo using MRI (N = 20) (~45 mm) [23] at the same angle. These differences may be associated with anthropometric scaling, with the average moment arm of children (37 mm) [23] and neanderthals (32 mm) [20], more comparable to the present results. Further, the TT projection index (length of the TT relative to tibial length) approximates the mechanical advantage of the quadriceps femoris [24], with an average value of 10.3 in human adults from a European population [20], whereas the average in this study was 17.9. These findings suggest a higher mechanical advantage within kangaroo stifles, however, the mechanisms for this superiority require further exploration and could be related to the increased projection of the TT, the relative dimensions of the femoral condyles, or the mismatch between tibial and femoral axial alignment. 

The specimen limb lengths, though comparable to previously reported data on *M. giganteus*, differed to that of humans [25]. The segments (400 mm) were comparable to Asian Indian data for males (355 mm) for the tibiae, whereas the kangaroo femur (200 mm) was much shorter than human males (425 mm) as reported by Pietak et al. [26]. This has important implications for muscle cross sectional area and volume. The CA.FL (product of the quadriceps cross-sectional area and femoral length) approximates the volume of the quadriceps muscles. In humans, this averages 29 cm^2^·m in men and 22 cm^2^·m in women [19] whereas it was lower in the present study (16.7 cm^2^·m), but higher than that of human children aged 6–9 (boys—9.4 cm^2^·m) [19]. Considering the considerably shorter femur in the specimens examined in this study, the differences in CA.FL may not be linear in scale. Therefore, the comparable torque generated at the knee requires further clarification in a dynamic model with realistic loading inputs. 

The limb segment and muscle profiles derived from the kangaroo stifles in this study confirm important structural differences between kangaroo and human knees, which need to be taken into account when drawing lessons for altering the biomechanical profile of the human knee in the context of injury and disease.

### 4.2. Functional Characteristics

The ability of the kangaroo stifle to achieve a considerable mechanical advantage without a patella provides some insight into options for modifying knee function in the context of PF disorders such as OA, instability and in extreme cases, resection. Previous studies examining the loading characteristics of the PF joint and its potential association with pain [27,28] and degeneration have identified a key role of the VM in determining loading patterns at the patella-trochlea cartilage interface [29]. While there has been some controversy regarding multiple portions of the VM in humans [30], the present results revealed a single muscle belly with similar origins to the human equivalent (inferior aspect of the lesser trochanter and medial linea aspera) and a similar insertion into the extensor tendon, inferior to the RF insertion [31]. 

Anteriorisation of the kangaroo stifle, as evident in the TP:TT ratio where the TT projection was up to half the size of the TP, is a structural feature prompting functional considerations for human knees. In one study, changes in patella kinematics were examined through realignment of the TT in humans and found that anteriorisation, in a manner that might replicate that of the kangaroo stifle, was associated with decreased patellar flexion during isometric loading of the knee at various degrees of knee flexion. Decreased lateral translation with medialisation of the tuberosity [32] was also found, but no relationship was found with patella tilt. Others have identified femoral and tibial rotation as potential factors associated with patellar stress during running [33,34]. A tibiofemoral rotational mismatch in the axial plane was also observed in the present study and may be a contributing factor to the extensor moment arm observed. 

Overall, the findings suggest that altering PF biomechanics, particularly in those requiring compensation for a reduced mechanical advantage, may be more complex than simply replicating prominent tuberosity as found in the kangaroo. Furthermore, when combined with architectural differences in the extensor musculature, these findings point to important functional differences in the use of the knee during locomotion that should also be considered in future investigations. 

### 4.3. Limitations

While the small sample size in the investigation yields a large range in the results, specifically in the limb lengths, flexion angles, QCA and pennation angles, the investigation serves the purpose of verifying that a static biomechanical profile can be generated with the methods described, and measures to quantify this profile can be obtained and compared to humans. Establishing the accuracy of these results will require repeat analyses, with a larger sample size. The lack of an intact hip joint on the specimens also meant that the origin points of the SA and RF could not be determined, thus it is recommended that specimens sourced for a dynamic analysis have a full hindlimb. Applied biomimetics is a multistage process, and this investigation lays the foundation upon which a more complex dynamic model can be developed, with the key structural and functional differences identified that need to be factored into future investigations and comparative human studies. In the long term, this line of investigation has the potential to provide deeper insights into the effect of anatomical variations on the biomechanical profile of the knee joint. Understanding these relationships is fundamental to inform treatment and management strategies for PFP and other knee disorders.

## 5. Conclusions

We aimed to establish metrics that could be quantified in the *M. giganteus* stifle, for the purpose of comparing them to the human knee in future dynamic analyses. The present study has quantified a biomechanical profile of the kangaroo stifle and revealed key structural and functional differences relative to the human knee. The anatomical arrangement of the stifle, lacking an ossified patella and its superior mechanical advantage compared to humans with a patella require further exploration. The data reported in this study can be used to inform the inputs and constraints of future comparative analyses from which important lessons can be learned for the human knee.

## Figures and Tables

**Table 1:** Static measurements of the kangaroo stifle.

	TL (mm)	FL (mm)	Flexion Angle (deg)	QCA (cm^2^)	QCA.FL (cm^2^·m)	Moment Arm (mm)	TT Projection Index	TP:TT RATIO	Pennation Angle (deg)	
									VL	RF
Limb 1	395.5	196.1	78.2	77.1	15.1	29.1	15.3	1.4	32.1	45.8
Limb 2	392.9	189.9	70.0	81.6	15.4	28.6	18.2	1.9	19.0	20.8
Limb 3	418.9	205.2	68.4	101.7	20.9	28.5	17.8	1.7	24.8	29.3
Limb 4	405.3	209.2	88.6	86.1	18.0	32.2	18.0	1.8	29.9	27.9
**Median**	**400.4**	**200.7**	**74.1**	**83.9**	**16.7**	**28.9**	**17.9**	**1.8**	**27.4**	**28.6**
**Range**	**26.0**	**20.1**	**20.2**	**24.6**	**5.8**	**3.8**	**2.9**	**0.5**	**13.1**	**25.0**

TL: tibial length; FL: femoral length; QCA: quadriceps cross-sectional area; QCA.FL: quadriceps cross-sectional area multiplied by femoral length; TT: tibial tuberosity; TP: tibial plateau; TT:TP ratio: tibial tuberosity to tibial plateau ratio.

**Table 2:** Regions of origin, knee extensor mechanism insertion points and femoral attachment of the key muscles.

Muscle	Origin	Insertion into Extensor Mechanism	Femoral Attachment
**SA**	Tuber coxae [18]	Most superficial insertion	None
**RF**	Lateral spine of the ilium cranial to the acetabulum [18]	Inferior to SA insertion	None
**VL**	Greater trochanter	Lateral insertion, inferior to RF	Greater trochanter only
**VI**	Greater trochanter	Inferior to VL and VM insertion	Continuous along the lateral aspect of femoral shaft
**VM**	Lesser trochanter	Medial insertion, inferior to RF	None, attached to medial intermuscular septum with VI

SA, sartorius; RF, rectus femoris; VL, vastus lateralis; VI, vastus intermedius; VM, vastus medialis.
